# Investigating How the Use of Technology Can Reduce Missed Appointments: Quantitative Case Study at a General Practitioner Surgery

**DOI:** 10.2196/43894

**Published:** 2024-07-29

**Authors:** Teresa Sides, Dhouha Kbaier

**Affiliations:** 1 The Open University Milton Keynes United Kingdom; 2 Glyndwr University Wrexham United Kingdom

**Keywords:** National Health Service, primary care, SMS text messaging, SMS reminders, missed appointments, quantitative research, Kruskal-Wallis test, Mann-Whitney test

## Abstract

**Background:**

SMS texting systems have been considered a potential solution to reduce missed appointments in primary care. Existing research in this area focuses on qualitative studies investigating the attitudes of SMS text users and receivers.

**Objective:**

This study aimed to examine appointment data from an independent general practitioner (GP) surgery in Wrexham, United Kingdom, with approximately 15,000 patients, to determine the impact of text messaging systems on reducing missed appointments. The objective of this study was to investigate whether the use of text messages can effectively reduce missed appointments.

**Methods:**

To collect data for the study, SQL reports were run on EMIS Web, the United Kingdom’s most widely used clinical system. The data spanned 10 years, from September 1, 2010, to March 31, 2020. Data accuracy was verified by cross-referencing with appointment diary records. Mann-Whitney and Kruskal-Wallis tests, chosen for their suitability in comparing groups in nonparametric settings, were conducted in Microsoft Excel due to its accessibility.

**Results:**

Statistical analyses were conducted to compare data before and after implementation of the text messaging system. The results revealed a significant 42.8% reduction in missed appointments (before: 5848; after: 3343; *P*<.001). Further analysis of demographic characteristics revealed interesting trends, with no significant difference in missed appointments between genders, and variations observed across different age groups. The median number of missed appointments was not significantly different between genders (women: 1.55, IQR 1.11-2.16; men: 1.61, IQR 1.08-2.12; *P*=.73). Despite the prevalence of mobile phone use among young adults aged 20-25 years, the highest rates of missed appointments (848/7256, 11.7%) were noted in this group, whereas the lowest rates were noted in the 75-80 years age group (377/7256; 5.2%; *P*<.001). Analysis by age and gender indicated inconsistencies: women aged 20-25 years (571/4216) and men aged 35-40 years (306/3040) had the highest rates of missed appointments, whereas women aged 70-75 years (177/4216) and men aged 75-80 years (129/3040) had the lowest rates (*P*<.001 for both).

**Conclusions:**

This study demonstrates that SMS text messaging in primary care can significantly reduce missed appointments. Implementing technology such as SMS text messaging systems enables patients to cancel appointments on time, leading to improved efficiency in primary care settings.

## Introduction

In recent years, primary care has adopted SMS text messaging systems for patient communication, yet their efficacy in achieving positive outcomes remains unknown. For general practitioner (GP) practices, the National Health Service (NHS) sets annual targets that determine their funding. Since 2019, in addition to clinical outcomes, there has been a shift toward enhancing accessibility and appointment availability [1].

The widespread use of mobile phones in daily lives across all age groups [2] has facilitated the development of cost-effective, bulk text messaging systems for businesses [3]. Text messages are universally compatible and accessible across various network speeds, and do not require upgrades [4]. While bulk text messaging has been widely explored in business, its applications and potential impact within health care remain underexplored, which is the focus of this study.

Numerous scholarly articles have delved into the perspectives of patients about text message reminders [5-7]. Sherman et al [8] found that while text messages were deemed useful, patients wanted the option to reply to the messages. Maslakpak and Safaie [9] compared treatment compliance between groups receiving text messages, reminder cards, or no reminders; they found no significant difference between reminder cards and text messages.

Hirst et al [10] reported that patients received a postal reminder with or without a text reminder (generated by the iPlato texting system [11] integrated with EMIS Web [12]). They reported a slight improvement in screening uptake from 39.9% to 40.5%, but the difference was not statistically significant.

In a study involving 18,138 participants, Fisher et al [13] evaluated the effectiveness and utility of text messages in health care, noting that text messages increased appointment attendance and patient satisfaction. However, nonrandom sampling may compromise validity. Since participants volunteered, their willingness to engage might influence appointment attendance, potentially introducing bias to the results.

Research on GPs using text messaging systems primarily investigates patient preferences for receiving texts and doctors’ willingness to send them [14]. The literature predominantly discusses the advantages and disadvantages of such systems from the GP standpoint, rather than assessing their impact on patient appointment attendance. Trials evaluating text reminder systems have yielded mixed results, with no significant overall improvement in attendance rates [5,6]. Even trials with substantial cohorts exhibited only a modest 0.6% attendance increase, suggesting limited benefits of using text reminders [13].

This study adds to the expanding knowledge base in the field and offers insights into implementing effective appointment management strategies in primary care.

## Methods

### Overview

This study analyzed missed appointment data from an independent GP practice in Wrexham, United Kingdom, spanning a decade (September 1, 2010, to March 31, 2020). Data were gathered from EMIS web, and the iPlato texting system was implemented on September 15, 2015. iPlato messages contained standard appointment information and allowed patients to respond or cancel the appointment. Before iPlato, patients often relied on appointment cards or memory. Approximately 74% of appointments were associated with a mobile number.

Data included adults aged 18-79 years, excluding children due to parental responsibility for missed appointments and older adults aged 80 years and above due to frailty or care home residence. Nurse data were not integrated into the system until September 2015, so only missed GP appointments were analyzed.

Missed appointments data over a decade were gathered using SQL reports to compare 5 years before and after text reminders were implemented. The collected figures were cross-referenced with appointment diary records to ensure accuracy.

Mann-Whitney and Kruskal-Wallis tests were used for quantitative analysis due to their suitability for nonparametric comparisons [15,16]. Mann-Whitney test, known for its efficiency and robustness [17], was used to assess gender categories, and Kruskal-Wallis test was applied across various age groups due to its suitability for comparing more than 2 groups.

### Ethics Approval

The Open University Human Research Ethics Committee approved this project (HREC/4180/Sides).

## Results

This study aimed to analyze missed appointment frequencies and demographic characteristics of patients who missed appointments. Of the 572,794 appointments booked, 9191 were missed, with 5286 (57.5%) attributed to women and 3905 (42.5%) to men (Table 1).

Mann-Whitney test results (Table 2) indicated higher rates of missed appointments before text messaging was implemented compared to after implementation, suggesting that text messaging can be an effective strategy for reducing missed appointments, particularly among women.

**Figure 1:**
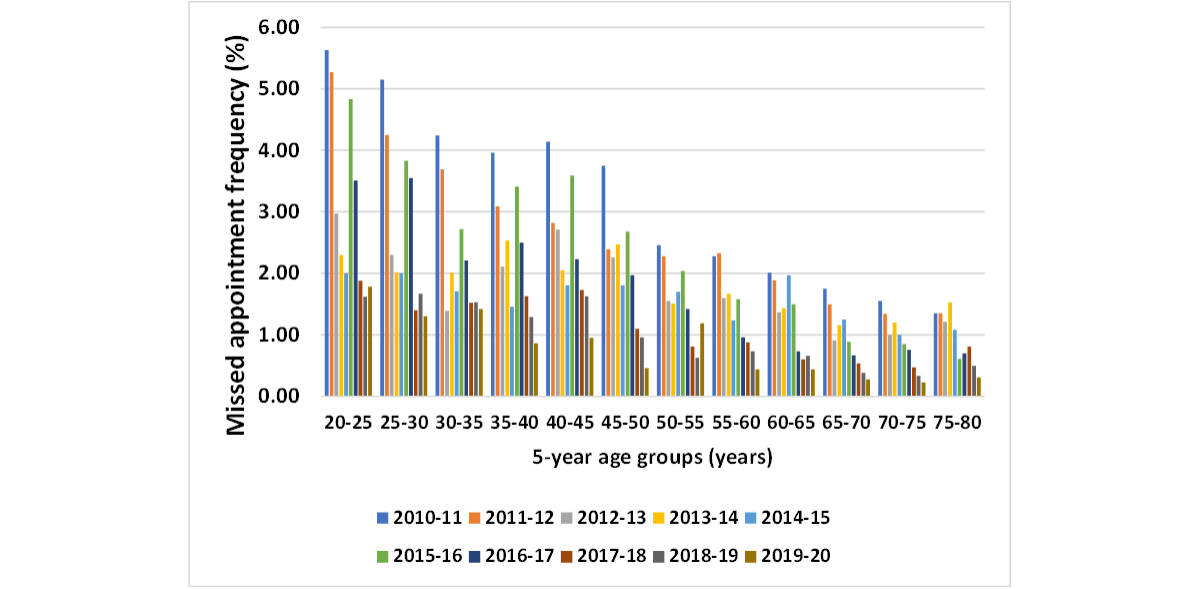
Missed appointment frequency across 5-year age groups.

To assess the data more accurately, missed appointment rates were calculated for each age group (Figure 1). Significant differences in median missed appointment numbers across different age groups were observed (Table 3). The 20-25 years age group had the highest median number of missed appointments, whereas the 65-80 years age group had the lowest. Further information and statistical data are available in Multimedia Appendix 1.

**Figure 2:**
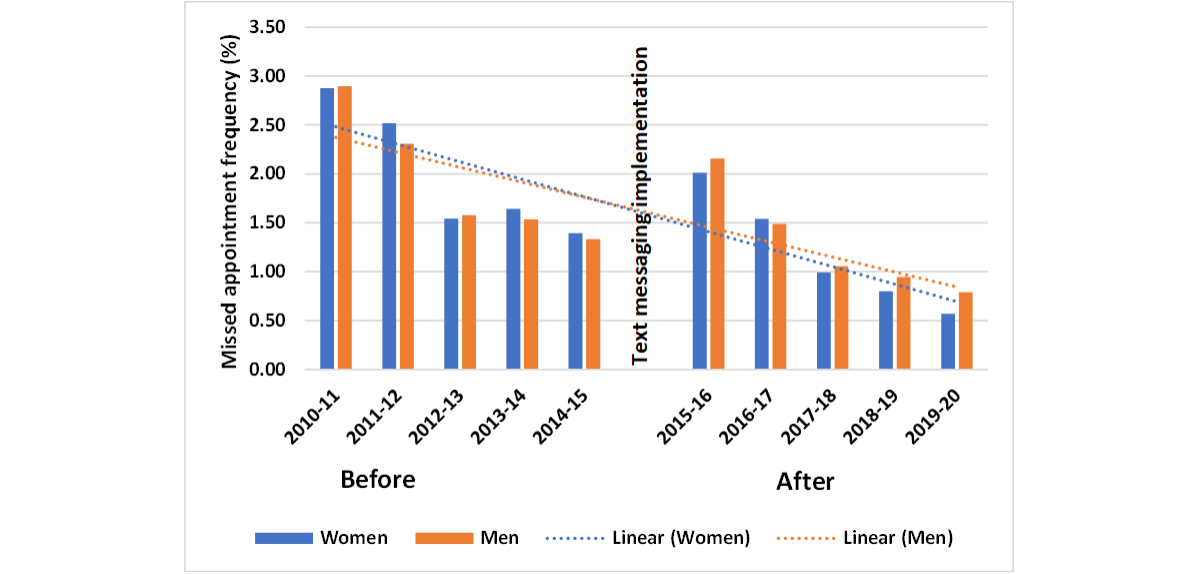
Missed appointment frequency by gender (with trend lines) before and after implementation of the SMS text messaging system.

Data analysis showed that text message reminders reduced missed appointments at the GP practice. Missed appointment frequency decreased before text messaging was introduced in September 2015 (Figure 2). Then, shortly after text messaging implementation, it increased, followed by a further decline to the lowest level noted in 10 years. Several factors may have contributed to this trend, including increased attention and improved data recording practices. Despite this factor, texting resulted in a statistically significant decrease in nonattendance at appointments (Table 1). These findings demonstrate the effectiveness of text messaging in reducing missed appointments.

**Table 1:** Population and gender descriptive statistics before and after text messaging implementation (N=9191 missed appointments).

Study Population		Missed appointments, n (%)			Reduction in missed appointments (%)
		Before text messaging implementation	After text messaging implementation		
**Total population**		5848 (63.6)	3343 (36.3)	42.8	
	Women	3435 (37.4)	1851 (20.1)	46.1	
	Men	2413 (26.3)	1492 (16.2)	38.2	

**Table 2:** Mann-Whitney test results (N=9191 missed appointments; 5286 by women and 3905 by men).

Patient population and frequency of missed appointments		Before text messaging implementation, median (IQR)	After text messaging implementation, median (IQR)	*U^a^*	*z* score	*P* value
**Total population**						
	Quarterly	1.8 (1.4-2.5)	1.1 (0.9-1.6)	74	3.1	.002
	Monthly	1.9 (1.5-2.7)	1.2 (0.8-1.6)	719	5.2	<.001
**Women**						
	Quarterly	1.9 (1.5-2.3)	1.1 (0.8-1.5)	72	3.2	.002
	Monthly	1.9 (1.5-2.5)	1.2 (0.7-1.6)	694	5.3	<.001
**Men**						
	Quarterly	1.8 (1.4-2.1)	1.1 (1.0-1.5)	80	2.9	.003
	Monthly	1.9 (1.5-2.5)	1.3 (1.0-1.7)	833	4.6	<.001

^a^Mann-Whitney U (Wilcoxon) statistic.

**Table 3:** Statistical results for missed appointments across 5-year age ranges (N=7256 missed appointments; 4216 by women and 3040 by men).

Study population and age range (years)			Patients, n (%)		*r*		Mean (SD)	s^2^		Median (IQR)
**Total population**										
	20-25	848 (11.7)		1.0		3.2 (1.5)		2.4	2.6 (1.9-4.5)	
	25-30	832 (11.5)		0.9		2.7 (1.3)		1.8	2.2 (1.8-3.8)	
	30-35	665 (9.2)		0.7		2.2 (1)		1.0	1.9 (1.5-2.6)	
	35-40	628 (8.7)		1.0		2.3 (1)		1.0	2.3 (1.5-3)	
	40-45	734 (10.1)		0.9		2.4 (0.9)		0.9	2.1 (1.8-2.8)	
	45-50	690 (9.5)		1.0		2.0 (0.9)		0.9	2.1 (1.3-2.5)	
	50-55	603 (8.3)		0.9		1.6 (0.6)		0.4	1.5 (1.3-2)	
	55-60	564 (7.8)		0.7		1.4 (0.6)		0.4	1.4 (0.9-1.7)	
	60-65	554 (7.6)		0.6		1.3 (0.6)		0.4	1.4 (0.7-1.8)	
	65-70	403 (5.6)		0.9		0.9 (0.5)		0.2	0.9 (0.6-1.2)	
	70-75	358 (4.9)		0.9		0.9 (0.4)		0.2	0.9 (0.5-1.2)	
	75-80	377 (5.2)		0.1		0.9 (0.4)		0.2	0.9 (0.6-1.3)	
**Women**										
	20-25	571 (13.5)		1.0		3.3 (1.6)		2.9	2.8 (2-4.9)	
	25-30	570 (13.5)		0.9		2.7 (1.3)		2	2.3 (1.8-3.6)	
	30-35	416 (9.9)		0.8		2.2 (0.9)		0.9	1.9 (1.6-2.4)	
	35-40	322 (7.6)		0.8		2 (0.9)		0.8	1.8 (1.4-2.5)	
	40-45	401 (9.5)		0.9		2.5 (1.5)		2.5	2 (1.4-3.5)	
	45-50	406 (9.6)		0.7		2 (0.9)		1	2.3 (1.5-2.5)	
	50-55	334 (7.9)		0.8		1.5 (0.6)		0.4	1.5 (1-2)	
	55-60	300 (7.1)		0.8		1.2 (0.6)		0.4	1.3 (0.7-1.7)	
	60-65	258 (6.1)		0.3		1 (0.5)		0.3	1.1 (0.7-1.3)	
	65-70	213 (5.1)		0.9		0.9 (0.5)		0.3	0.9 (0.4-1.2)	
	70-75	177 (4.2)		0.9		0.8 (0.4)		0.2	0.9 (0.5-1)	
	75-80	248 (5.8)		0.2		1.3 (1.2)		1.5	1.1 (0.7-1.3)	
**Men**										
	20-25	277 (9.1)		0.7		3 (1.3)		1.9	2.5 (2-3.8)	
	25-30	262 (8.6)		0.9		2.7 (1.3)		1.8	2.4 (1.7-3.7)	
	30-35	249 (8.2)		0.5		2.3 (1.1)		1.2	1.7 (1.5-2.9)	
	35-40	306 (10.1)		0.9		2.8 (1.2)		1.7	2.6 (1.5-3.8)	
	40-45	333 (11)		0.5		2.5 (0.7)		0.6	2.4 (2.1-2.7)	
	45-50	284 (9.3)		0.8		1.9 (1)		1.1	1.9 (1.2-2.5)	
	50-55	269 (8.8)		0.9		1.8 (0.6)		0.4	1.8 (1.4-2.1)	
	55-60	264 (8.7)		0.7		1.5 (0.7)		0.5	1.4 (1-2.1)	
	60-65	296 (9.7)		0.9		1.4 (0.7)		0.5	1.7 (0.7-1.9)	
	65-70	190 (6.3)		0.9		0.9 (0.4)		0.1	0.8 (0.7-1)	
	70-75	181 (6)		0.9		0.9 (0.5)		0.3	0.9 (0.6-1.2)	
	75-80	129 (4.2)		0.4		0.8 (0.3)		0.1	0.8 (0.6-1.1)	

## Discussion

This study explores the impact of SMS text messaging systems on reducing missed appointments in primary care, focusing on a single GP practice. While a significant 42.8% decrease in missed appointments was noted after implementation of text messaging reminders, caution is warranted in generalizing these results to broader health care settings. This 42.8% reduction is significant when compared with the findings of Maslakpak and Safaie [9] who found no significant difference.

Age group analysis revealed interesting findings regarding missed appointments. Surprisingly, the 20-25 age group emerged as significant overall, with a higher percentage of missed appointments, contrary to expectations given their high mobile phone use. De-Sola et al [18] observed increased phone use for the 16-25 years age group. However, despite prolific phone usage, the 20-25 years age group patients did not exhibit reduced missed appointments. These findings highlight the positive impact of text reminder systems on missed appointments overall, yet further exploration is needed to understand factors influencing attendance in specific demographics.

In conclusion, text messaging reminders can effectively reduce missed appointments, enabling more GP consultations. However, it is unlikely that missed appointments can be completely avoided. Health care technology offers opportunities for further improvement, including timely text reminders and systematic patient data maintenance. By embracing technology in health care, we can overcome challenges, maximize appointment availability, and enhance patient care.

## Appendix

Multimedia Appendix 1 Study data and Mann-Whitney and Kruskal-Wallis test results.

## Abbreviations

GP: general practitioner
NHS: National Health Service

## Data Availability

All data generated or analyzed during this study are included in Multimedia Appendix 1.
